# The effects of clinical and pharmacological factors on the ratio of clozapine to norclozapine in psychiatric patients

**DOI:** 10.3389/fphar.2024.1518739

**Published:** 2025-01-07

**Authors:** Anna Mach, Anna Wnorowska, Marcin Siwek, Marcin Wojnar, Maria Radziwoń-Zaleska

**Affiliations:** ^1^ Department of Psychiatry, Medical University of Warsaw, Warsaw, Poland; ^2^ Department of Affective Disorders, Jagiellonian University Medical College, Cracow, Poland; ^3^ Department of Psychiatry, Addiction Center, University of Michigan, Ann Arbor, MI, United States

**Keywords:** clozapine, norclozapine, clozapine-to-norclozapine ratio, clozapine metabolism, schizophrenia

## Abstract

**Background:**

Due to its exceptional effectiveness, clozapine (CLO), whose metabolite is norclozapine (NCLO), is a drug of choice in the management of treatment-resistant schizophrenia. The purpose of this study was to assess the factors modifying the CLO/NCLO ratio (CNR).

**Methods:**

A total of 446 blood samples (233 of which were drawn from females and 213 from males, aged from 18 to 77 years) were analyzed in this study. The patients were treated at a psychiatric hospital in the period 2016–2021. Serum CLO and NCLO levels were determined with high-performance liquid chromatography coupled with a UV detector.

**Results:**

The median CNR was 2.38 (minimum 0.30, maximum 14.36). Our analysis showed that neither sex (p= 0.135) nor smoking (*p* = 0.774) had any significant effect on the CNR. However, increased doses of CLO resulted in lower CNR values (*p* = 0.005). Concomitant use of other psychotropic drugs increased the CNR (*p* = 0.001).

**Discussion:**

The results of our study indicate a need for personalized CLO treatment. Assessing the CNR may be useful in identifying CLO interaction with other psychotropic drugs.

## 1 Introduction

Clozapine is an atypical antipsychotic drug considered to be the most effective in treating treatment-resistant schizophrenia (Siskind et al., 2016). Meta-analyses showed that the use of CLO reduces the rates of hospitalization and treatment discontinuation (Land et al., 2017; Masuda et al., 2019). Continuous treatment for many years may decrease mortality and prolong the life of patients with treatment-resistant schizophrenia (van der Zalm et al., 2021; Cho et al., 2019). Moreover, irrespective of its effect on other symptoms, CLO decreases suicidal and aggressive behaviors (Meltzer et al., 2003; Citrome and Volavka, 2021).

Apart from CLO affinity to dopaminergic receptors D1 and D2 and serotoninergic receptors 5-HT2A, the pharmacological profile of the drug additionally includes an affinity to adrenergic receptors α1 and α2A–C, histamine receptor H1, serotoninergic receptors 5-HT2B and 5-HT2C, and muscarinic receptors M1–4 (Ellison and Dufresne, 2015). However, the specific mechanisms of action responsible for CLO’s superior effectiveness remain unexplained (de Bartolomeis et al., 2022). The spectrum of action and the risk of certain adverse drug reactions associated with CLO have been attributed to its main active metabolite N-desmethylclozapine (or norclozapine, NCLO) (Costa-Dookhan et al., 2020a). NCLO is characterized by a more potent antagonist action on 5-HT1C, 5-HT2C, D1, and D2 receptors than the original compound. The unique clinical efficacy of CLO and NCLO is due to the combination of actions on very numerous receptor systems. In addition to the aforementioned receptors, CLO also shows activity against receptors: D3, D4, 5-HT1A, 5-HT1B, 5-HT1D, 5-HT1E, 5-HT3, 5-HT6, and 5-HT7 (Knox et al., 2024). The main mechanism associated with CLO-induced weight gain and metabolic disorders is believed to be the potent effect of NCLO on receptor 5-HT2C (Lu et al., 2004a; Mendoza and Lindenmayer, 2009). Moreover, CLO and NCLO exert opposite effects on the cholinergic system. NCLO is a full or partial agonist of M1, M3, M4, and M5 receptors, which is unique for an antipsychotic drug (Molins et al., 2017). CLO and its active metabolite have been observed to have different effects in the hippocampus and medial prefrontal cortex, which are areas involved in cognitive functions, including executive functions and memory (Melancon et al., 2013). Therefore, NCLO is expected to have pro-cognitive potential (Tarek et al., 2015). Moreover, NCLO’s full agonism towards M1 receptors and its partial agonism towards M3 receptors (which outweighs CLO’s antagonism towards M3 receptors) may explain the increased salivation observed during CLO use (Ishikawa et al., 2020; Chen et al., 2019). NCLO is produced as CLO is metabolized in the liver, mostly via cytochrome P450 1A2 (CYP1A2) and, to a lesser extent, CYP3A4, CYP2C19, CYP2C9, and CYP2D6 (Khokhar et al., 2018). Modulating the CLO/NCLO ratio (CNR) by targeting CYP enzymes may modify the resultant receptor profile of CLO (Couchman et al., 2010).

In order to optimize treatment, serum CLO and NCLO levels should be monitored in a process referred to as therapeutic drug monitoring (Hiemke et al., 2018). Due to considerable interpersonal variability in CLO metabolism, therapeutic drug monitoring is the basis for personalized dosing (Raedler et al., 2008; Piwowarska et al., 2016). Serum NCLO levels are believed to fall somewhere between 20% and 150% of serum CLO levels (Bondesson and Lindström, 1988). The normal therapeutic ranges of CLO and NCLO for the lowest steady-state drug concentration are 350–600 ng/mL and 100–600 ng/mL, respectively (de Leon et al., 2020; Patteet et al., 2014; Hiemke et al., 2018). However, the optimum CNR value with respect to adverse drug reactions (including granulocytopenia), the pharmacological profile of CLO, or CYP1A2 activity has not been established; nor has its significance as a possible predictor of cognitive functions or working memory been determined (Ellison and Dufresne, 2015; Couchman et al., 2010; Polcwiartek and Nielsen, 2016; Molins et al., 2017; Sarpal et al., 2022). Moreover, the CNR may play an important role in estimating the risk of pharmacokinetic interactions due to the drug’s complex hepatic metabolism (Siwek et al., 2020; Siwek, 2015).

Studies showed that higher CNR values were associated with better cardiometabolic outcomes and a potentially better tolerance of CLO therapy. Conversely, lower CNR values were associated with better cognitive function (Costa-Dookhan et al., 2020a; Islam et al., 2020), although these results were not consistent (Arnautovska et al., 2021). The CNR may be useful in predicting and monitoring cardiometabolic adverse effects of CLO and in optimizing the drug’s potential pro-cognitive benefits. In one study, the CNR was modified pharmacologically to reduce the side effects and increase the effectiveness of therapy in treatment-resistant schizophrenia. Combining CLO with fluvoxamine, which is a CYP 1A2 inhibitor, helped lower NCLO levels, effectively increasing the CNR, which improved treatment effectiveness and limited certain side effects (such as: sedation, weight gain, metabolic disturbances, and neutropenia) (Légaré et al., 2013b). Nonetheless, we cannot recommend this method due to the fact that CLO may potentially reach toxic levels, resulting in complications (Siwek, 2015). Given the paucity of data, it is not recommended to manipulate the CNR to reduce adverse drug reactions or improve cognitive functions (Meyer, 2023). Therefore, further studies are needed to identify the correlations between CLO and NCLO levels.

Most studies on this topic assessed neither the CNR nor factors that might affect its value. Moreover, previous empirical studies estimated neither the therapeutic nor toxicity index of CLO. Discovering interpersonal variation in the CNR and in the factors that affect its value would help better understand CLO metabolism and its potential clinical implications.

## 2 Materials

A total of 446 serum CLO and NCLO test results were included in this study. The test results were obtained from patients aged from 18 to 77 years (mean age 47.4 ± 14 years), treated at the Independent Regional Complex of Public Psychiatric Healthcare Facilities in Warsaw in the period between year 2016 and 2021.

Out of the analyzed test results, 233 (52.2%) were from females and 213 (47.8%) were from males. The vast majority of the analyzed test results (n = 428, 96.0%) were from patients diagnosed with schizophrenia. The remaining test results were from patients with schizoaffective disorders (n = 9, 2.0%), mental disorders due to brain damage or dysfunction (n = 6, 1.3%), or acute and transient psychotic disorders (n = 3, 0.7%).

Out of the analyzed test results, 241 (54.0%) were from smokers, 155 (34.8%) from nonsmokers, and 50 (11.2%) were from individuals with an unknown smoking status.

## 3 Methods

This study was approved by the local Ethics Committee at the Medical University of Warsaw (approval No. AKBE/83/2021).

All patients whose blood tests were analyzed had been receiving daily CLO doses appropriate for their clinical status. Demographic data and other information, including the daily dose of CLO, smoking status, and other concomitant drugs were obtained from the patients’ medical records and referrals for therapeutic drug monitoring issued by psychiatrists. Only blood tests that were from patients hospitalized in closed wards were analyzed, which largely eliminated cases of poor adherence to treatment or active abuse of psychoactive substances.

The diagnoses of schizophrenia, schizoaffective disorders, mental disorders due to brain damage or dysfunction, and acute and transient psychotic disorders were established based on the International Classification of Diseases – 10th Revision (ICD-10) of the World Health Organization (World Health Organization, 1993), ICD-10 codes F20, F25, F06, and F23, respectively.

The therapeutic ranges from 350 to 600 ng/mL for CLO and from 100 to 600 ng/mL for NCLO were adopted according to Arbeitsgemeinschaft für Neuropsychopharmakologie und Pharmakopsychiatrie (AGNP) guidelines (Hiemke et al., 2011; Hiemke et al., 2018; de Leon et al., 2022). The toxicity level for CLO, defined as the level with an increased risk of drug toxicity, was adopted at ≥1,000 ng/mL (Hiemke et al., 2018). We adopted the recommended daily dose of CLO between 200 and 450 mg based on European Medicines Agency (EMA) guidelines (EMEA, European agency for the evaluation of medicinal products, 2002).

All blood samples analyzed in this study for serum CLO and NCLO levels had been collected from patients at a steady state (after at least 7 days of continuous stable dosing) and at trough concentration (samples collected 10–14 h after the last dose).

CLO and NCLO levels were determined via high-performance liquid chromatography (HPLC), with a Shimadzu chromatograph with a UV detector. The method has been described in detail in an earlier paper (Mach et al., 2024).

The statistical analysis of quantitative variables was conducted with the use of descriptive statistics, such as means, standard deviations, medians, and ranges. The Shapiro–Wilk test was used to test whether the distribution of the analyzed quantitative variables deviated from a normal distribution. If the analyzed distribution was normal, we used the *t*-test to verify the hypothesis of equality of the means for two groups; if the distribution was not normal, we used the nonparametric tests to compare independent groups, the Wilcoxon Rank Sum test for two and the Kruskal–Wallis test for more groups. Spearman correlations were used to measure the association between pairs of variables.

Relationships between categorical variables were evaluated with the use of contingency tables and the chi-square test or Fisher’s exact test for small sample sizes.

The logistic regression generalized linear model (GLM) was employed in multivariate analysis. The optimal model was selected based on the Akaike information criterion (AIC) statistic. Type III tests were used to calculate the significance of each of the effects specified in the model.

P-values of less than 0.05 were considered statistically significant.

Statistical analysis calculations were conducted with the use of SAS/STAT v.15.2.

## 4 Results

Table 1 presents CNR, CLO and NCLO concentrations, daily CLO dose, and CLO concentration-to-dose ratio.

**TABLE 1:** Clozapine-to-norclozapine ratio (CNR), clozapine (CLO) and norclozapine (NCLO) levels, daily CLO dose, and CLO concentration-to-dose (C/D) ratio.

	N	Mean	SD	Median	Minimum	Maximum
CNR	446	2.53	1.10	2.38	0.30	14.36
CLO levels [ng/mL]		508.9	319.4	453.5	42.0	1,753.0
NCLO levels [ng/mL]		220.0	154.2	176.5	31.0	1,161.0
Daily CLO dose [mg]		378.2	151.8	375.0	100.0	900.0
CLO C/D ratio (ng/ml per mg/day)		1.49	1.03	1.20	0.19	5.87

The CNR showed a positive correlation (r = 0.17, p< 0.001) with blood CLO levels, and a negative correlation (r = −0.33, p< 0.001) with NCLO levels.

The daily CLO dose (r = −0.13, p = 0.006) showed a negative correlation with the CNR.

The median CNR was 2.38 (min. 0.30, max. 14.36). The median CNR in samples from patients with a CLO concentration within the therapeutic range (350–600 ng/mL) was 2.32 (min. 1.18, max. 14.36). The median CNR in samples from patients with a NCLO concentration within the therapeutic range (100–600 ng/mL) was similar at 2.31 (min. 0.30, max. 5.46). The CNR values for three concentration ranges (therapeutic, subtherapeutic, and supra-therapeutic) showed significant differences for CLO (p = 0.004) and NCLO (p< 0.001) (Table 2).

**TABLE 2:** Clozapine (CLO) and norclozapine (NCLO) levels and the clozapine-to-norclozapine ratio.

	Clozapine-to-norclozapine ratio						
	N	Mean	SD	Median	Minimum	Maximum	p-value
CLO levels [ng/mL]							
<350	160	2.36	0.88	2.26	0.30	6.10	0.004
350–600	161	2.53	1.25	2.32	1.18	14.36	
>600	125	2.75	1.13	2.56	1.06	11.06	
<1,000	408	2.51	1.12	2.33	0.30	14.36	0.102
≥1,000	38	2.71	0.88	2.60	1.16	4.95	
NCLO levels [ng/mL]							
<100	94	3.02	1.74	2.77	0.98	14.36	<0.001
100–600	339	2.43	0.81	2.31	0.30	5.46	
>600	13	1.68	0.47	1.51	1.06	2.56	

There was no significant difference in the CNR value between the blood samples with toxic (≥1,000 ng/mL) and nontoxic (<1,000 ng/mL) CLO levels (p = 0.102).

Table 3 shows the CNR stratified by the daily CLO dose.

**TABLE 3:** The subtherapeutic, therapeutic, and supra-therapeutic clozapine dose ranges and the corresponding clozapine-to-norclozapine ratios.

Daily CLO dose (mg)	Clozapine-to-norclozapine ratio						
	N	Mean	Standard deviation	Median	Minimum	Maximum	p-value
<200	46	2.79	1.92	2.54	0.30	14.36	0.012
200–450	284	2.58	1.02	2.46	0.98	11.06	
>450	116	2.30	0.79	2.18	1.05	6.10	

The median CNR in the 284 blood samples from patients receiving CLO at therapeutic doses (200–450 mg/day) was 2.46 (min. 0.98; max. 11.06). A comparison of the three ranges of daily CLO doses (subtherapeutic, therapeutic, and supra-therapeutic) presented in Table 3 revealed a significant difference in the CNR (p = 0.012).

Patient sex (p= 0.135) and smoking status (p = 0.774) did not affect the CNR. Detailed data have been presented in Table 4.

**TABLE 4:** The clozapine-to-norclozapine ratio in females and males and in smokers and nonsmokers.

	Clozapine-to-norclozapine ratio						
	N	Mean	Standard deviation	Median	Minimum	Maximum	p-value
Females	233	2.53	0.80	2.44	1.06	5.88	0.135
Males	213	2.54	1.36	2.25	0.30	14.36	
Nonsmokers	155	2.52	0.93	2.45	1.05	5.88	0.774
Smokers	241	2.58	1.24	2.36	0.30	14.36	

In our study, the age of the patients whose blood samples were analyzed showed a positive correlation with the CNR (r = 0.10; p = 0.034).

Out of all analyzed blood samples, 262 came from patients who were receiving other drugs in conjunction with CLO (other psychotropic drugs, n = 251; beta-blockers, n = 44; antidiabetic drugs, n = 15; and angiotensin converting enzyme inhibitors, n = 7). The concomitant psychotropic drugs included (in the order of decreasing rates): valproic acid, aripiprazole, amisulpride, hydroxyzine, haloperidol, clorazepate, risperidone, chlorprothixen, venlafaxine, diazepam, lithium, levomepromazine, pregabalin, sertraline, flupentixol, sulpiride, sertraline, olanzapine, fluoxetine. There were also single patients (<1%) who were receiving mirtazapine, mianserin, clomipramine, citalopram, alprazolam, zuclopenthixol, escitalopram, paroxetine, perazine, nordiazepam, or lamotrigine. The beta-blockers included nebivolol, bisoprolol, propranolol, atenolol, and metoprolol. The remaining 184 blood samples came from patients who were receiving CLO in monotherapy. The median CNR in samples from patients who were receiving CLO in monotherapy was 2.31 (min. 1.05; max. 4.74). The median CNR in samples from patients who were receiving CLO in conjunction with other drugs was 2.46 (min. 0.30; max. 14.36). The CNR value was significantly lower in blood samples from patients treated with CLO only (p = 0.009).

No significant modulatory effect on the CNR was observed when analyzing blood samples from patients who were additionally on beta-blockers (p = 0.147), antidiabetic drugs (p = 0.399), or angiotensin-converting enzyme inhibitors (p = 0.140).

We analyzed the effect of psychotropic drugs received in combination with CLO.

The CNR values in patients receiving CLO as the only psychotropic drug (n = 195; median 2.35; min. 1.05; max. 4.74) and in those concomitantly receiving other psychotropic drugs (n = 251; median 2.45; min 0.30; max. 14.36) differed significantly (p = 0.017).

Out of all analyzed blood samples, there is a very low representation of determinations from patients who did not receive a psychotropic drug as an additional treatment (n = 11).

The optimal model of a multivariate analysis to assess factors affecting the CNR includes the following variables: CLO dose and the use of additional psychotropic drugs (Table 5).

**TABLE 5:** Type III significance tests for variables included in the model for clozapine-to-norclozapine ratio.

Type III tests of effects		
Effect	F Value	p-value
Daily clozapine dose	7.87	0.005
Additional psychotropic drugs	10.78	0.001

**FIGURE 1:**
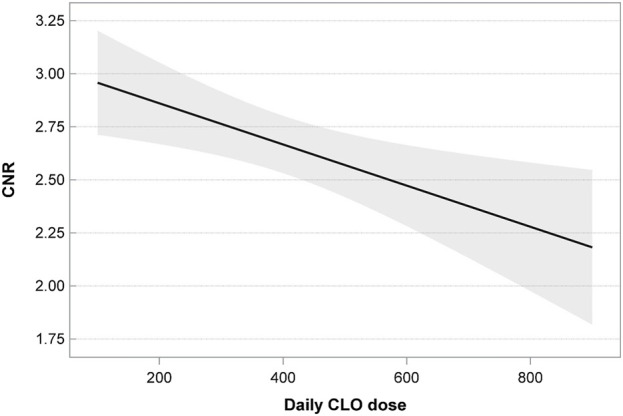
The CNR value and the CLO dose.

**FIGURE 2:**
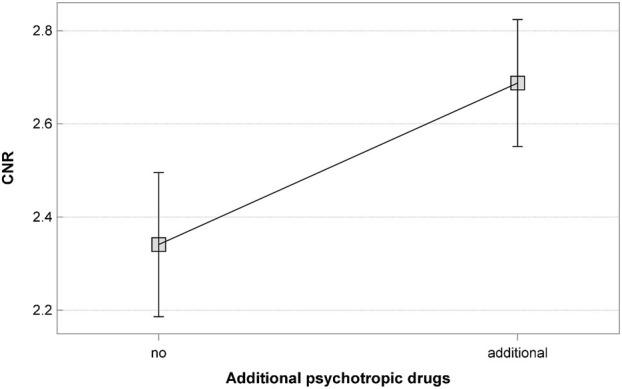
The effect of receiving additional psychotropic drugs, apart from CLO, on the CNR.

The presented model showed the effects of the following on the CNR:

• The CLO dose; the linear relationship of the CNR and CLO dose was statistically significant (p = 0.005); as the dose increased, the value of CNR decreased as well (Figure 1).
• The use of additional psychotropic drugs; blood samples from patients receiving CLO in monotherapy showed significantly lower CNR values (p = 0.001) (Figure 2).

The analyzed data showed two CNR values that were extremely low (0.3; 1.0) and 5 that were extremely high (5.5; 5.9; 6.1; 11.1; 14.1). One of these blood samples (CNR = 5.9) came from a woman, whereas the remaining ones came from men. Table 6 presents additional data on extreme CNR values.

**TABLE 6:** Cases of extreme CNR values.

CNR	CLO levels [ng/mL]	NCLO levels [ng/mL]	Daily CLO dose [mg]	Age	CLO C/D ratio (ng/ml per mg/day)	Sex	Smoking	Drugs other than CLO	Suspected causes
0.3	42	137	175	62	0.24	M	Yes	Amisulpride, valproic acid	An inhibitory effect of valproate on UGT; smoking
1.0	65	66	200	29	0.33	M	Yes	Levomepromazine, nebivolol	UM; impaired renal elimination of NCLO; levomepromazine induces CYP2D6; smoking
5.5	989	181	300	49	3.30	M	No	Olanzapine, amisulpride, hydroxyzine	Blocking of CYP1A2 by olanzapine; CLO and olanzapine competing for CYP1A2 and CYP2D6
5.9	365	62	350	52	1.04	F	No	Aripiprazole	Blocking of CYP2D6 by aripiprazole; an inhibitory effects of estrogens on CYP1A2
6.1	348	57	600	61	0.58	M	Yes	Valproic acid, clorazepate	Blocking of CYP 1A2, 3A4, and 2C19 by valproate
11.1	686	62	450	36	1.52	M	Yes	Valproic acid	Blocking of CYP 1A2, 3A4, and 2C19 by valproate
14.1	474	33	125	63	3.79	M	Yes	Risperidone, ramipril	Blocking of 2D6 by risperidone; competing with CLO for CYP 3A4

C/D ratio, clozapine concentration-to-dose ratio; CLO, clozapine; CNR, clozapine-to-norclozapine ratio; NCLO, norclozapine; F, female; M, male; UGT, UDP-glucuronyltransferase; UM, ultrarapid metabolizer.

## 5 Discussion

The analyzed blood samples (n = 446) yielded a median CNR of 2.38 (min. 0.30; max. 14.36, mean 2.53 ± 1.1). These results are consistent with the mean CNR values of 2.4–2.6 (n = 14) reported by other authors (Murayama-Sung et al., 2011), 2.44 ± 1.03 (n = 32) (McArdle et al., 2019) and with its reported median values of 2.2 ± 0.6 (n = 39) (Iglesias et al., 2017). Some authors reported lower values of CNR. In a systematic review that included analysis of blood samples from 2,317 adult patients from 19 different trials Schoretsanitis et al. reported a mean CNR value of 1.73, with a range from 1.19 to 3.37 (Schoretsanitis et al., 2019). The median CNR values in blood samples from 26,796 inhabitants of Great Britain and Ireland in relation to plasma concentration of CLO were 1.25 at < 350 ng/mL, 1.55 at 350–600 ng/mL, 1.78 at 610–1,000 ng/mL, and 2.08 at >1,000 ng/mL (Couchman et al., 2010). Wickramarachchi et al., who analyzed blood samples from 247 patients, reported a median CNR of 1.54 (range 1.23–1.99) (Wickramarachchi et al., 2022). Rostami-Hodejegen et al. obtained a mean CNR of 1.32 from 3,782 blood samples (Rostami-Hodjegan et al., 2004).

The differences in CNR values mentioned above may be due to study sample selection as well as CNR-modifying factors. The CNR value may provide information on CLO metabolism. Since the elimination half-life of NCLO is longer than that of CLO, lower CNR values may suggest a rapid metabolism of CLO or patient non-adherence over the previous 24 h (Ellison and Dufresne, 2015; Couchman et al., 2010). In our study we analyzed only blood samples obtained from inpatients, which practically eliminates the likelihood of non-adherence. This may be one of the reasons why we achieved a slightly higher median CNR than other authors.

A CNR of at least two has been suggested to increase CLO treatment effectiveness and tolerance (Légaré et al., 2013a), at the same time indicating that CLO metabolism has become saturated (Couchman et al., 2010). Most authors suggested that an increased CNR was associated with a better clinical response to CLO treatment and/or side effect reduction (Lu et al., 2000; Lu et al., 2004a). Grover et al. adopted a cutoff CNR value of 1.54. Using this cutoff value, the authors identified the patients responding to CLO treatment and differentiated those patients from non-responders with a 86% sensitivity (Grover et al., 2022). However, studies on this topic are not consistent. There have been reports of a positive correlation between low CNR values and the clinical response (Weiner et al., 2004; Sporn et al., 2007).

A handful of studies produced a mean CNR value higher than that obtained in our study. A mean CNR value of 2.7 ± 1.1 (0.8–8.8) (n = 77) was achieved in patients concomitantly receiving fluvoxamine (Shymko et al., 2018). This is due to the potent inhibitory effect of fluvoxamine on CLO metabolism; therefore, international guidelines recommend avoiding this drug combination (de Leon et al., 2022). At the same time, a high CNR value (of >3) suggests that the drugs used in conjunction with CLO may inhibit CLO metabolism (Couchman et al., 2010). A study by Flanagan et al. showed that the median CNR in the case of a non-lethal CLO overdose was 7.6 (5.3–18.0) (Flanagan et al., 2005a). This indicates that the CNR may provide additional information on a CLO overdose or interactions.

We achieved very similar median CNR values for therapeutic levels of CLO (2.32; min. 1.18; max. 14.36) and NCLO (2.31; min. 0.30; max.5.46). Interestingly, a comparison of CNR values in three ranges of CLO and NCLO levels (therapeutic, subtherapeutic, and supratherapeutic) yielded statistically significant differences both for CLO and NCLO. The observed increase in CNR, which corresponds to higher CLO levels, may reflect a saturated metabolism of the drug.

One factor that can independently modify the CNR value is the daily CLO dose. Our previous study demonstrated a positive correlation between the daily CLO dose and both CLO and NCLO levels. This correlation was linear and was statistically significant (Mach et al., 2024). In our present study we observed that the daily CLO dose correlated negatively with the CNR. The median CNR obtained from the blood tests of patients receiving the recommended CLO doses (200–450 mg/day) was 2.46 (min. 0.98; max. 11.06; mean 2.58). An increase in the daily dose of CLO resulted in decreased CNR values. CLO doses exceeding 450 mg/day produced a median CNR value of 2.18 (min. 1.05; max. 6.1; mean 2.30), whereas doses below 200 mg/day produced higher CNR values than recommended doses. Importantly, there were significant differences in the CNR values between patients receiving therapeutic, subtherapeutic, and supratherapeutic doses.

Recent years saw the use of CLO concentration-to-dose (C/D) ratio in CLO TDM. This ratio is a measure of CLO clearance, which may affected by genetic or environmental factors. The CLO C/D ratio may be calculated with the use of CLO levels only or the sum of CLO and NCLO levels, which helps achieve a more personalized CLO dosing. CLO levels of >150 ng/mL are believed to produce a linear and stable C/D ratio (de Leon, 2022). However, there have been reports of situations in which the use of CLO C/D ratio analysis is limited, particularly in the case of potential drug interactions. An inverted CNR (<1) was a more sensitive indicator than the C/D ratio of inhibited renal elimination of NCLO by gemfibrozil (Barclay et al., 2019).

Patient sex was repeatedly reported to be an important factor modifying blood CLO and NCLO levels (Liu et al., 2023; Mach et al., 2024; Mayerova et al., 2018). Higher blood CLO levels in females may be explained by a lower CYP1A2 activity, inhibited both by estrogens and oral contraceptives (Bigos et al., 2009). Moreover, in comparison with men, women have a greater proportion of fat tissue, which tends to accumulate CLO due to its lipophilicity (Diaz et al., 2018). The CNR obtained in our study showed no association with patient sex. This result was consistent with those reported in earlier studies and meta-analyses by other authors (Schoretsanitis et al., 2019; Palego et al., 2002; Iglesias et al., 2017; Lee et al., 2009).

Smoking status is known to considerably affect the metabolism of CLO (Mach et al., 2024; Wagner et al., 2020). The lower blood levels of CLO and NCLO observed in smokers are likely due to CYP1A2 activation, induced by aromatic carbohydrates contained in tobacco smoke (Cormac et al., 2010; Brownlowe and Sola, 2008). Our study showed no significant differences in CNR values in blood samples from smokers and those from nonsmokers. This result is consistent with those reported by some other authors (Wagner et al., 2020; Palego et al., 2002; Tang et al., 2007; Schoretsanitis et al., 2019), although not all studies yielded equally consistent results (Lozano and Bona, 2023). The observed lack of significant effect of both smoking and sex on the CNR seems to indicate that CNR values are unlikely to be a strong marker of CYP1A2 activity. This conclusion is consistent with that from pharmacogenetics studies, which revealed that—unlike the CLO C/D ratio—variants of the CYP1A2 gene had no significant association with the CNR (Melkersson et al., 2007).

Another factor that modifies blood CLO and NCLO levels is concomitant use of other drugs (Mach et al., 2024). The CNR in our study was significantly higher in blood samples from patients concomitantly receiving other drugs, particularly other psychotropic drugs. On the one hand, higher CNR values may be a result of the additional drugs impeding CLO metabolism by inhibiting CYP450, particularly CYP1A2 (de Leon et al., 2022). Concomitant use of multiple drugs may potentiate this inhibitory effect. On the other hand, some studies suggest that although the CNR provides information on CLO distribution and clearance, it does not directly reflect CYP1A2 activity, despite the considerable role of this isoform in CLO metabolism (Schoretsanitis et al., 2020; Schoretsanitis et al., 2019). Apart from undergoing metabolism by CYP, an estimated 83% of NCLO is excreted by the kidneys via the P-glycoprotein (P-gp) transporter (Schoretsanitis et al., 2019). Both P-gp function and expression, like those of CYP, are subject to population variation, which affects NCLO clearance (Schmitt et al., 2012; Meyer, 2023). The increase in CNR observed in patients receiving other psychotropic drugs in addition to CLO suggests CYP inhibition or impaired NCLO excretion develops without significant changes in CLO levels. Higher CNR values may also indicate a more severe patient’s condition, requiring treatment with higher doses of CLO, in conjunction with other drugs. In patients requiring treatment with higher doses of CLO, higher CNR values may also be due to saturated metabolism of CLO (Couchman et al., 2010).

Seven of the analyzed blood samples yielded extremely low or extremely high CNR values. Three of those samples came from patients concomitantly receiving valproic acid, which is one of the factors whose effects on CLO metabolism are of clinical significance. Due to individual variations, valproic acid may act either as an inducer or an inhibitor (Wilkowska et al., 2023). Although the exact mechanism of this interaction is unknown, it is suspected to involve CLO metabolism inhibition as well as NCLO metabolism induction (de Leon, 2020). Therefore, it may either decrease or increase the CNR value. Another extremely high CNR value was observed in a patient concomitantly receiving risperidone. Although combined use of CLO and risperidone may be beneficial in some treatment-resistant cases, it requires careful assessment and consideration. There have been reported cases of CLO levels being considerably increased in response to combination therapy with risperidone (Tyson et al., 1995). Concomitant use of CLO and olanzapine may similarly lead to increased CLO levels. Both drugs are metabolized primarily by CYP1A2, therefore olanzapine may inhibit CLO metabolism by this isoenzyme (Gee et al., 2015). Another extremely high CNR value was observed in a patient concomitantly receiving aripiprazole. Despite a lack of documented important pharmacokinetic interactions between these two drugs, isolated studies revealed a possible increase in CLO levels due to aripiprazole (Reactions weekly, 2016; Zink, 2007). There have been also reports of cases in which discontinuation of levomepromazine resulted in a four- or tenfold increase in CLO levels (Bugamelli et al., 2007). Levomepromazine is thought to induce CYP2D6, which takes part in CLO metabolism (Hals and Dahl, 1994). This assumption may explain the extremely low CNR values in samples from patients receiving levomepromazine and smoking, which activates CYP1A2. Despite the fact that valproic acid, risperidone, olanzapine, levomepromazine, and aripiprazole may to some extent modify the CNR, they do not belong to drugs producing the greatest clinically significant interactions with CLO.

The phenomenon of NCLO levels exceeding CLO levels (CNR<1) was defined as an inverted CNR (Ruan et al., 2024). In our study, the lowest CNR value was 0.3, which was similar to those reported by other authors (0.3; 0.5; 0.5) (Wickramarachchi et al., 2022; Couchman et al., 2010; Patteet et al., 2014). One cause of an inverted CNR may be a genetically determined ultrarapid metabolizer phenotype or impaired renal elimination of NCLO (Flanagan et al., 2023). A CNR of ≤0.5 was described as an indicator of ultrarapid metabolism of CLO (Flanagan et al., 2023); however, this was disputed by Schoretsanitis et al. (Schoretsanitis et al., 2024). It is worth noting that a low CNR may be also a predictor of adverse drug reactions. Those of NCLO, such as excessive salivation, constipation, sedation, myoclonus, seizures, or weight gain may manifest more strongly in patients with low CNR values (McCollum et al., 2018; Ohno-Shosaku et al., 2011; Sur et al., 2003). Wickramarachchi et al. emphasized that the CNR is stable over time in the steady state, even if CLO and NCLO levels fluctuate. As a result, patients with a low CNR may never achieve clinical effectiveness of CLO treatment without experiencing adverse drug reactions induced by NCLO (Wickramarachchi et al., 2022). According to a review of literature by Ruan et al. the rates of inverted CNR ranged from 0.8% to 24.7%. In some cases this phenomenon could not be explained (Ruan et al., 2024).

Reverse situations, in which CLO levels considerably exceed those of NCLO leading to extremely high CNR values, may be due to suppressed N-demethylation of CLO by other concomitantly used drugs or concomitant systemic inflammation (Flanagan et al., 2023). These situations may be also due to a genetically determined poor metabolizer phenotype or CYP450 gene polymorphism (Milosavljevic et al., 2021; Caetano and Piatkov, 2015). We obtained five high CNR values; these ranged from 5.3 to 18.0, a range reported by Flanagan et al. in patients with suspected CLO poisoning as a result of an overdose (Flanagan et al., 2005b).

Extreme CNR values may be also due to patient nonadherence although inpatient treatment reduces that risk. Nonetheless, extremely low CNR values responsible for an inverted CNR may suggest that CLO had not been taken for a day or two before the blood sample was collected for testing. Due to a longer elimination half-life of NCLO, it takes a longer time for NCLO levels than for CLO levels to decrease after CLO discontinuation (Schoretsanitis et al., 2019; Ruan et al., 2023). Moreover, extremely high CNR levels may suggest that the patient has not been taking CLO regularly (Flanagan et al., 2023).

It is wort noting that in the case of most of the obtained extreme CNR values the C/D ratio also exceeded the values expected. For the recommended daily doses of CLO between 200 and 450 mg the C/D ratio is expected to remain in the range of 1.40–0.88 (Wilkowska et al., 2023). Only in a single case of a high CNR value was the C/D ratio within the expected range, which may suggest that the CNR has higher sensitivity than the C/D ratio in cases of drug interactions.

Our study had several limitations. Our analysis of CNR did not include the renal function of patients whose blood samples were analyzed. NCLO is excreted by proximal tubules and expelled with urine (Schaber et al., 1998). Although routine lab tests, such as serum creatinine levels and estimated glomerular filtration rate (eGFR) are markers of glomerular filtration and do not directly show proximal tubule function, these tests have been shown to be associated with NCLO levels and the CNR (Schoretsanitis et al., 2019). We are unable to exclude potential effects of other confounding factors, such as inflammation, caffeine, obesity, or pharmacogenetics factors (Pfuhlmann et al., 2009). This paper does not present the effect of the individual drugs administered concomitantly with CLO; this aspect will be presented in detail in a subsequent paper. The blood samples analyzed in this study came from Polish citizens. In light of ethnicity-based variations in CLO metabolism, it may not be advisable to extrapolate our conclusions onto Asian populations or native inhabitants of the Americas (de Leon et al., 2022). Importantly, our analysis was conducted in a closed hospital setting, where drug administration is overseen by medical personnel. The rate of nonadherence in other, similar research models was approximately 1% (Ruan et al., 2020; Schoretsanitis et al., 2019).

The results of our study show the need for a personalized approach to CLO treatment. CNR value assessments may provide additional clinical information. Changes in CNR may be a warning to the clinician that the patient had been exposed to CYP inhibitors or inducers, discontinued or resumed treatment, or showed nonadherence in the use of other drugs that inhibit or induce CYP. In the case of considerably higher or lower CNR values in patients receiving CLO in monotherapy, diagnostic assessments should be expanded to include assessing the function of the organs involved in metabolism of the drug and the presence of certain gene variants, for example, CYP450 variants (Caetano and Piatkov, 2016). Future studies are necessary to substantiate our study results and to evaluate whether the CNR value correlates with symptom improvement or side effects. Expanding our knowledge on the clinical usefulness of CNR may play an important role in treatment optimization.

## 6 Conclusion

1) The following parameters affected the CNR: the dose of the drug and concomitant use of other psychotropic drugs.
2) The CNR ratio decreases with increasing clozapine dose.
3) The CNR value may indicate interactions between CLO and other psychotropic drugs.
4) Extreme CNR values may occur irrespective of the CLO dose.
5) The CNR is not associated with patient sex.
6) Smoking does not affect the CNR.

## Data Availability

The raw data supporting the conclusions of this article will be made available by the authors, without undue reservation.
